# How a few poorly designed COVID-19 studies may have contributed to misinformation in Brazil: the case for evidence-based communication of science

**DOI:** 10.1136/bmjos-2021-100202

**Published:** 2021-09-02

**Authors:** Charles Phiilipe de Lucena Alves, João de Deus Barreto Segundo, Gabriel Gonçalves da Costa, Tatiana Pereira-Cenci, Kenio Costa Lima, Flávio Fernando Demarco, Inácio Crochemore-Silva

**Affiliations:** 1 Federal University of Pelotas, Pelotas, Brazil; 2 Bahiana School of Medicine and Public Health, Salvador, Brazil; 3 Federal University of Rio de Janeiro, Rio de Janeiro, Brazil; 4 Federal University of Rio Grande do Norte, Natal, Brazil

**Keywords:** press release, misinformation, COVID-19, COVID-19 pandemic

## Introduction

The emergence of SARS-CoV-2 in the end of 2019, an aetiologic agent responsible for the 1SARS plunged the world into an unprecedented sanitary crisis. Papers on COVID-19 have been fast-tracked since then.^2–5^ Accelerated time from submission to publication^6–8^ and qualitative changes in peer review,^9^ associated with empirical evidence that duplicate and implausible clinical trials have been carried out during the pandemic,^10–12^ could perhaps imply lower quality of peer review in COVID-19 research.

Accumulating empirical evidence has also been indicating the pandemic era output to be less reliable than its prepandemic counterpart.^2 10–14^ A systematic review to evaluate the methodological quality of COVID-19 peer-reviewed clinical studies compared with historical controls found methodological quality scores to be lower in COVID-19 articles across all study designs.^12^ Meanwhile, data sharing practices remained largely unchanged during the first year of the pandemic.^13 14^ With no mandates of data sharing in place for COVID-19 studies, the reproducibility of these data on COVID-19 is yet to be independently verified as well.^2^ However, more efficiency in scientific publication did manifest in accelerated publication,^6–8^ journals tearing down their paywalls for their COVID-19 output,^14^ an increased usage of life and medical sciences preprint servers to increase speed and transparency,^15^ not to mention the intense international collaboration that resulted in the development of multiple high-efficacy vaccines within the first year of the pandemic.^16^

On the other hand, some pratices that reduce the reliability of clinical trials may have gained some traction during 2020, such as executing underpowered studies with small samples, multiplicity of trials testing ideas with low prior probability of being true, forgoing blinding to test interventions^10 11 14 17–20^ and incomplete reporting of findings, which was already an issue before the pandemic.^12 21^ To what extent that has dominated the general output in medical interventions for COVID-19 and how much of it turned into actual clinical pratice is something that has not yet been thoroughly assessed and is, thus, still open for debate.^11 14^ Notwithstanding, it is likely that poor science, even if it being the exception within an overall output, when carelessly amplified within a context of sanitary crisis and political polarisation, may be consequential, as it has been the case of the now infamous hydroxychloroquine (HCQ) study,^22^ that strengthened a trend in non-evidence-based interventions for COVID-19 and divided the Brazilian medical community to this date.^23 24^ The mechanism of how that type of misinformation plays out in the current media environment is the topic of interest of this brief communication as disseminating scientific findings through press releases and press conferences but without timely access to the study nor to its data has also gained some traction in 2020 in Brazil.^13 14 25^

Below, we briefly discuss how a small set of exceptionally poorly designed studies disseminated through the press, weeks or months before publication and without access to the data sets used to generate these studies for a more thorough assessment, played into a cycle of misinformation in Brazil in the first year of the pandemic. And, to conclude, we suggest a programme of scientific investigation aimed to properly examine and address those issues and foster trust between scientific community, the media and society.

## The Brazilian case scenario

The attempt to repurpose an already known and readily accessible drug for the treatment of COVID-19 started early in the pandemic with a piece published in preprint format about HCQ,^23^ a drug already safely deployed in the treatment of a set of autoimmune diseases and malaria for decades. A few days later, the preprint appeared as published paper, virtually unchanged, in the International Journal of Antimicrobial Agents,^22^ a publication of the International Society of Chemotherapy. It is noteworthy that, by the time the authors wrote this brief communication, in the journal editorial board, three of the coauthors of the HCQ paper sat, one as editor-in-chief. Later on, the paper earned an expression of concern by the society, yet no retraction followed.^26^ The paper ended up being discussed in the media and HCQ started being promoted as early treatment for COVID-19 in the USA and in Brazil by their presidents, respectively, strengthening a worldwide trend in non-evidence-based interventions for COVID-19.^19 20 23 24^ Both societies were then very politically polarised already.

A few weeks later, a Brazilian study reported positive effects of HCQ and azithromycin usage on suspected early stage COVID-19 cases. The study was posted as a Dropbox file, then widely circulated through WhatsApp among physicians, scientists and journalists.^27–29^ It is very hard to assess how widely it was distributed, but it reached the national press, the national medical community and the international scientific community. A press release was also sent out by the authors to journalists reporting the study findings and they conceded interviews to discuss their claims. To the best of our knowledge, the study was never uploaded to any preprint server and never underwent review in any journal. It presented major issues, easy to spot during an actual in-depth peer review: (a) no patients were randomised, (b) patients with influenza-like symptoms were included without COVID-19 testing for confirmation of what disease was being treated, (c) on average, the treatment group had more symptoms when compared with the control group, (d) reasons for hospitalisations and deaths were not disclosed; (e) the significance could not be as high as the authors claimed it to be, as the p values did not match the data disclosed. Worst of all, (f) ethical aspects were violated as patients were allocated and treated before ethical clearance.^28 29^

The HCQ narrative offered hope packed in a cheap, readily available solution to the sanitary crisis before people would get sick and die. It was a perfect fit into a stereotyped narrative of science, one that has been time and again criticised for being detached from the laborious way science actually works.^30 31^ Even after the Brazilian mainstream media picked up the pace and tried to disavow HCQ based on actual science, the story had already gained a life of its own, eclipsing science-based medicine.^23 24^ The Brazilian Federal Council of Medicine, the class association that grants physicians their licenses and regulates their practice in the country, as of June 2021, still has not publicly disavowed HCQ as early intervention for COVID-19. Therefore, physicians are still allowed to dispense the drug after patient or family consent, however, wasteful that may be from a scientific standpoint.

### The ivermectin spin-off

The antiparasitic drug ivermectin for treating COVID-19 appeared first in the scientific literature in an in vitro study. The study conclusion, sensibly, stated the inherent limitations of the in vitro model.^32^ The drug was also investigated in a later retracted observational study, posted to Social Science Research Network, that claimed lower hospital mortality when observing patients who received the drug. That study also displayed restraint in its conclusions, cautioning that conclusive findings would require controlled settings in randomised trials.^33^ Equivalent results were also found in another observational study, with similar limitations and statements about the unfeasibility of extrapolation of those findings.^34^ In the meantime, building policy on observational studies, some Latin American governments, including Brazil, embraced ivermectin as a potential treatment for COVID-19, stating in press conferences that the drug would be added to their guidelines of COVID-19 prevention and treatment. At the same time, social distancing and mask wearing were discouraged publicly by the Brazilian federal administration, feeding into a false sense of security by means of non-evidence-based prophylaxis. At the time, clinical trials in the region struggled to recruit participants to test the efficacy of the drug against COVID-19 because people were already taking it.^35^

In January 2021, a randomised clinical trial was published demonstrating that ivermectin had no effect on the proportion of patients with detectable SARS-CoV-2 RNA by PCR from nasopharyngeal swab at day 7 post-treatment regimen.^36^ Also in early 2021, authors from the Front Line COVID-19 Critical Care Alliance had a manuscript accepted for publication in Frontiers in Pharmacology claiming efficacy of ivermectin against COVID-19, which was retracted shortly afterwards, before definitive publication. However, by the time it was retracted, in March 2021, its abstract had already been viewed over 85 thousand times.^37^

Much like a poorly written sequel to a blockbuster, the ivermectin narrative appears to be a subsidiary of the rationale that gave the world the HCQ pseudo-solution to COVID-19: cheap, readily available answer to the biggest sanitary crisis of our time. Following this logic, even with low prior probability, if an antiparasitic drug could help (it could not), perhaps another could help too.

### The nitazoxanide spin-off

Nitazoxanide, another antiparasitic drug, was tested both in vitro and in humans for efficacy against COVID-19 in a state-sponsored trial in Brazil.^38^ The federal government conveyed a press conference and sent out press releases claiming the drug was effective in reducing the viral load when taken within 3 days after the onset of symptoms. The press conference took place before the paper was available for public scrutiny.^39^ Four days after the press conference, the study appeared in the preprint platform medRxiv, in October 2020,^40^ showing a non-significant difference in its primary outcome: symptom resolution in patients with mild COVID-19. But viral load was a secondary outcome in the study,^38^ being a surrogate outcome that may not have clinical importance.

Spinning the study results in that fashion characterises selective reporting, a questionable practice in scientific communication. It can hinder or distort the translation of research into clinical practice and, when reproduced by the media, it can provide unrealistic expectations about new treatments and about the scientific enterprise, feeding distrust and misguided choices.^41 42^ It is also noteworthy that the peer-reviewed version of paper on nitazoxanide, which was published only in December 2020, differs next to nothing from its early preprint version.^38 40^

The nitazoxanide spin signals a strong pull towards forcing a poorly designed and conducted study to fit into a narrative of hope already sedimented into public discourse at the time.^35^ Three antiparasitic drugs underwent repurposing attempts to no avail and under no clear probable rationale other than being of low cost of production, readily available. Maybe it could be a case of the association fallacy by way of the ‘argumentum ad populum’: when many people have been doing something, as it appears popular, many others will try and do it as well, regardless of adherence to facts or reason). But that is not how science is supposed to work.^2 10 11 13 14 17–20 25 29–31^

## Discussion

Broader scientific dissemination and transparency are core ethical values of contemporary science, and interactions with the press should aim to build trust in the scientific method by increasing scientific literacy. The reliability of the scientific enterprise itself leans on its capacity to publicly demonstrate empirical findings, so that claims may be independently verified by scientists, independent press and concerned citizens.^43 44^ Evidence indicates that people generally display solidarity in crises and that unified authoritative messages may be consequential to achieving behaviour change,^45^ and thus compliance to sanitary measures for COVID-19 prevention, such as physical distancing and mask-wearing mandates. Before vaccines started being deployed, those non-medical courses of intervention were critical COVID-19 mitigation strategies in the long run.

Press releases are themselves unified authoritative messages intended to frame a coverage in a particular way, usually sent out from a press office of an institution to journalists operating in newspapers, news hub or agencies and television or radio stations. They comprise a narrative contextualising a given subject for reporting by the journalists, presenting a brief description of a problem, context, methods, main findings and its implications to the scientific community and to a broader audience. Although the press release format has been around since the beginning of the 20th century, only in the past two decades it has become a part of scientific communication landscape, being used, before the pandemic, usually by universities and scientific journals.^46 47^

The adoption of press releases for science communication happened in conjunction with profound changes in the news media ecosystem brought about by the widespread commercial use of the Internet at the end of the 20th century. Media corporations had been cutting jobs in their newsrooms while having their ranks of specialised journalists substituted by generalist professionals who could cover a multitude of topics at the expense of depth in order to lower production costs. That, in turn, resulted in an even greater demand for press releases, research spokepersons and scientific sources to help journalists translate scientific jargon into comprenhesible socially responsible coverage.^47 48^ Pressures of the market, thus, placed a lot of pressure both on the scientific sources and on the press to maintain an accurate coverage. That requires a delicate system of checks and balances from both the scientific community and the press. Additionally, there has been accumulating evidence that it is the knowledge of how science works and not the access to scientific facts and consensuses that is associated with persuasion and long-lasting behaviour change in polarised contexts.^49^

### The case for evidence-based communication of science

In the past three decades, media studies have established framing as a set of phenomena in which the journalistic coverage reduces some elements of the perceived reality. Then, the coverage arranges the remaining essential items into a coherent narrative that promotes an interpretation anchored on plausibility. This process tends to, in itself, tone down nuance and simplify context, which are essential features to the accurate reporting of findings in scientific papers. Framing in news coverage generally encompasses four functions: (a) problem definition, (b) causal analysis, (c) moral judgement and (d) the promotion of solutions. The framing is also expected to cue, shape and change the interpretations of the readers and audience members as well as their preferences, introducing or increasing the apparent importance of certain ideas, activating mental schemes that encourage the audience to think, feel and decide in a particular way.^50–53^ Ideally, a press release stemming from the scientific community would uphold all that four functions but also regard accuracy and as much context from the scientific paper as the press release format constraints would allow. That is particularly relevant because media studies have also been demonstrating framing to not yield strong lasting results in the public due to many other cognition processes related to self-perception, perception of others and expectation to belong to preferred groups and identities.^16 49 52 54–64^

Self-perception and group belonging may also happen at the expense of facts and rational decision-making and even promote risk-taking behaviour should that be a condition to maintain one’s identity and one’s ability to pertain to an identarian group.^54 55 57 60 61 64^ That phenomenon has been referred to in past research as pluralistic ignorance^58^ and, more recently, as motivated reasoning^64^ and functions under the following rationale: if the social cost to be persuaded may be the compromise of one’s perception of themselves, of their identity and ideology, and if those are key to in-group belonging as they offer a shared worldview by means of heuristics, one may prefer to not adhere to facts nor to scientific consensuses, instead cherry-picking aspects that fit into an interpretation of the reality in alignment with the one provided by the group. That happens possibly because to our ancestors being isolated from the peers, that is, their group, would have been a tangible existential threat.^16 45 49 64^ In other words, a shared sense of self is essential to cognition and behaviour, both being highly social processes in our species.^45^

Then, what happens when poorly designed COVID-19 studies get the spotlight through press conferences and press releases without checks and balances—such as study and data availability? Following the theoretical framework presented here, it is likely that national independent press would have covered the topic with the necessary degree of scepticism and nuance, consulting with independent scientific sources for critical assessment and that is something that should be empirically verified. Following the same rationale, partisan press would have the studies framed to fit into a particular worldview already promoted beforehand by those same outlets. The press conferences and releases would have served merely as amplifying tools, meanwhile the poorly designed studies, disseminated before the rest of the scientific community could verify and discredit them, would provide a scientific coating to a political stance already available through partisan coverage in some Brazilian press outlets. The difference in coverage in light of partisanship media outlet should also be empirically verified to test that hypothesis.

Following this rationale, in a glass half-full interpretation, those poorly designed studies alone would not have caused misinformation but surfed and gained momentum in an undertow already present in Brazilian society in the past few years, the same undertow that elected a president who has been ignoring and denying scientific evidence.^65 66^ However, the glass half-empty interpretation would signal that there is much to be done yet regarding scientific literacy in Brazil as only knowledge on how science works has been demonstrated to yield some power against identarian heuristics or worldviews and the unscientific polarisation they are associated with.^49^ Empirical research and the best evidence-based practices in science communication could perhaps foster scientific literacy through the common ground of a shared sense of self, which would in the long run help turn the tide of science denial.^16 45 49^ That is yet to be empirically verified as well, but it is very much aligned to the scientific ethos and fits the theoretical framework presented herein.^43 44^

We emphasise that any interaction between scientists and press should have aimed to summarise and contextualise the most important findings of an article for the general public, preserving context and limitations of the research, promoting transparency, integrity and scientific literacy. But that cannot be achieved if the promoted studies are faulty. And one cannot assess that, either in the scientific community or in the press if the paper takes weeks or months to be posted to a preprint server or published in a journal. One cannot assess that either if the preprint or the paper is not accompanied by their data sets.^13 14^ Otherwise, those interactions may be only fueling polarisation, which may be associated with the eventual implementation of harmful, inefficient or wasteful public health policies.

Therefore, we suggest a programme of scientific inquiry into associations between study methodological quality, science communication practices, media frames (taking into consideration outlet partisanship or lack thereof) and estimated impact on public behaviour in the context of the COVID-19 pandemic. Another necessary research pathway would be to try and understand which medical practices stemmed from poorly designed research on COVID-19, the cost of that in medical resources and in avoidable deaths. Transdisciplinary research connecting the biomedical sciences and the social sciences could help foster this research agenda and promote this debate.

## Conclusion

Science is an endeavour prone to failure as it should be. It is hardly about discovery of hidden truths and mostly about lowering uncertainty about the natural world as to maybe extrapolate general laws from findings, thus generating theories and predicting nature whenever possible to the common benefit of society regardless of political ideology. However, as scientists must press for more nuance in the media coverage, they also must not give into questionable research practices, questionable research report practices nor questionable public science communication practices. Public communication of science should be evidence based as well, and there is much to learn from the current crisis.

## Data Availability

Data sharing not applicable as no datasets generated and/or analysed for this study. No data are available.

## References

[1] Esakandari H, Nabi-Afjadi M, Fakkari-Afjadi J, et al. A comprehensive review of COVID-19 characteristics. Biol Proced Online 2020;22:19. 10.1186/s12575-020-00128-2 32774178 PMC7402395

[2] Correia LC, Barreto Segundo JdeD. An immunization program against the COVID-19 infodemic. Journal of Evidence-Based Healthcare 2020;2:7–9. 10.17267/2675-021Xevidence.v2i1.3124

[3] Dinis-Oliveira RJ. COVID-19 research: pandemic versus "paperdemic", integrity, values and risks of the "speed science". Forensic Sci Res 2020;5:174–87. 10.1080/20961790.2020.1767754 32939434 PMC7476615

[4] Palayew A, Norgaard O, Safreed-Harmon K, et al. Pandemic publishing poses a new COVID-19 challenge. Nat Hum Behav 2020;4:666–9. 10.1038/s41562-020-0911-0 32576981

[5] Else H. How a torrent of COVID science changed research publishing - in seven charts. Nature 2020;588:553. 10.1038/d41586-020-03564-y 33328621

[6] Helliwell JA, Bolton WS, Burke JR, et al. Global academic response to COVID-19: cross-sectional study. Learn Publ 2020;33:385–93. 10.1002/leap.1317 PMC7362145 32836910

[7] Horbach SPJM. Pandemic publishing: medical journals strongly speed up their publication process for COVID-19. Quant Sci Stud 2020;1:1056–67. 10.1162/qss_a_00076

[8] Barakat AF, Shokr M, Ibrahim J. Timeline from receipt to online publication of COVID-19 original research articles. MedRxiv 2020.

[9] Horbach SPJM. No time for that now! qualitative changes in manuscript peer review during the Covid-19 pandemic. Res Eval 2021:rvaa037. 10.1093/reseval/rvaa037

[10] Glasziou PP, Sanders S, Hoffmann T. Waste in covid-19 research. BMJ 2020;369:m1847. 10.1136/bmj.m1847 32398241

[11] Janiaud P, Axfors C, Van't Hooft J, Hooft J, et al. The worldwide clinical trial research response to the COVID-19 pandemic - the first 100 days. F1000Res 2020;9:1193. 10.12688/f1000research.26707.2 33082937 PMC7539080

[12] Jung RG, Di Santo P, Clifford C, et al. Methodological quality of COVID-19 clinical research. Nat Commun 2021;12:943. 10.1038/s41467-021-21220-5 33574258 PMC7878793

[13] Larregue J, Vincent-Lamarre P, Lebaron F. COVID-19: where is the data? 2020. LSE Blog. Available: https://blogs.lse.ac.uk/impactofsocialsciences/2020/11/30/covid-19-where-is-the-data/

[14] Moher D. COVID-19 and the research scholarship ecosystem: help! J Clin Epidemiol 2021;137:133–6. 10.1016/j.jclinepi.2021.03.032 33892088 PMC8455105

[15] Fraser N, Brierley L, Dey G, et al. The evolving role of preprints in the dissemination of COVID-19 research and their impact on the science communication landscape. PLoS Biol 2021;19:e3000959. 10.1371/journal.pbio.3000959 33798194 PMC8046348

[16] Correia LCL, Barreto Segundo JdeD. Are some physicians afraid of the COVID-19 vaccines? A tale of motivated Reasoning. Journal of Evidence-Based Healthcare 2020;2:117–24. 10.17267/2675-021Xevidence.v2i2.3813

[17] Pacheco RL, Martimbianco ALC, Latorraca CdeOC, et al. Why COVID-19 trials should be blinded (as any other one). Journal of Evidence-Based Healthcare 2020;2:25–7. 10.17267/2675-021Xevidence.v2i1.2841

[18] Pacheco RL, Martimbianco ALC, Ferreira RES, et al. Ensaios clínicos são mais difíceis de serem conduzidos em COVID-19 do que em outras condições? Journal of Evidence-Based Healthcare 2020;2:35–7. 10.17267/2675-021Xevidence.v2i1.3010

[19] Chaves AFA, Nogueira F. Potential treatments for COVID-19 are not parachutes – avoiding a pandemic of medical reversals. Journal of Evidence-Based Healthcare 2020;2:48–50. 10.17267/2675-021Xevidence.v2i1.3058

[20] Lemos Correia LC, Mandrola J. Do pandemics justify non-evidence-based therapies? Journal of Evidence-Based Healthcare 2020;2:51–3. 10.17267/2675-021Xevidence.v2i1.3125

[21] Tressoldi PE, Giofré D, Sella F, et al. High impact = high statistical standards? Not necessarily so. PLoS One 2013;8:e56180. 10.1371/journal.pone.0056180 23418533 PMC3571951

[22] Gautret P, Lagier J-C, Parola P, et al. Hydroxychloroquine and azithromycin as a treatment of COVID-19: results of an open-label non-randomized clinical trial. Int J Antimicrob Agents 2020;56:105949. 10.1016/j.ijantimicag.2020.105949 32205204 PMC7102549

[23] Nogueira F, Brito JC. The costly hydroxychloroquine battle in Brazil. Journal of Evidence-Based Healthcare 2020;2:45–7. 10.17267/2675-021Xevidence.v2i1.3054

[24] Correia LC, Lopes JRP, Garcez FB, et al. Physicians’ preference towards the non-evidence based hydroxychloroquine treatment for COVID-19: the pandemic effect. Journal of Evidence-Based Healthcare 2020;2:10–15. 10.17267/2675-021Xevidence.v2i1.3014

[25] Breznau N. Science by press conference: what the Heinsberg study on COVID-19 demonstrates about the dangers of fast, open science, 2020. LSE Blog. Available: https://blogs.lse.ac.uk/impactofsocialsciences/2020/08/20/science-by-press-conference-what-the-heinsberg-study-on-covid-19-demonstrates-about-the-dangers-of-fast-open-science/

[26] Marcus AA. Hydroxychloroquine-COVID-19 study did not meet publishing society’s “expected standard”, 2020. Available: https://retractionwatch.com/2020/04/06/hydroxychlorine-covid-19-study-did-not-meet-publishing-societys-expected-standard/ [Accessed 25 Feb 2021].

[27] Esper RB, da Silva RS, Oikawa FTC. Empirical treatment with hydroxychloroquine and azithromycin for suspected cases of COVID-19 followed-up by telemedicine [circulated on WhatsApp®. unreferenced] 2020.

[28] Bik E. Thoughts on the prevent senior study, 2020. Available: https://scienceintegritydigest.com/2020/04/18/thoughts-on-the-prevent-senior-study/

[29] Orsi C, Pasternak N. Uma aula de como não se deve testar um medicamento Revista Questão de Ciência. Available: https://www.revistaquestaodeciencia.com.br/questao-de-fato/2020/04/18/uma-aula-de-como-nao-se-deve-testar-um-medicamento

[30] Santos BS. Da sociologia da ciência política científica. Revista Crítica de Ciências Sociais 1978;1:11–56.

[31] Santos BS. Um discurso sobre as ciências. São Paulo, SP: Cortez, 2009.

[32] Caly L, Druce JD, Catton MG, et al. The FDA-approved drug ivermectin inhibits the replication of SARS-CoV-2 in vitro. Antiviral Res 2020;178:104787. 10.1016/j.antiviral.2020.104787 32251768 PMC7129059

[33] Patel A, Desai S. Ivermectin in COVID-19 related critical illness. SSRN Electronic Journal 2020. 10.2139/ssrn.3570270

[34] Rajter JC, Sherman MS, Fatteh N, et al. Use of ivermectin is associated with lower mortality in hospitalized patients with coronavirus disease 2019: the ivermectin in COVID nineteen study. Chest 2021;159:85–92. 10.1016/j.chest.2020.10.009 33065103 PMC7550891

[35] Mega ER. Latin America's embrace of an unproven COVID treatment is hindering drug trials. Nature 2020;586:481–2. 10.1038/d41586-020-02958-2 33077974

[36] Chaccour C, Casellas A, Blanco-Di Matteo A, et al. The effect of early treatment with ivermectin on viral load, symptoms and humoral response in patients with non-severe COVID-19: a pilot, double-blind, placebo-controlled, randomized clinical trial. EClinicalMedicine 2021;32:100720. 10.1016/j.eclinm.2020.100720 33495752 PMC7816625

[37] Offord C. Frontiers removes controversial Ivermectin paper pre-publication, 2021. Available: https://www.the-scientist.com/news-opinion/frontiers-removes-controversial-ivermectin-paper-pre-publication-68505

[38] Rocco PRM, Silva PL, Cruz FF. Early use of nitazoxanide in mild Covid-19 disease: randomised, placebo-controlled trial. Eur Respir J 2021;57:2003725. 10.1183/13993003.03725-2020 PMC7758778 33361100

[39] *G1*. Ministério da Ciência diz que vermífugo ajuda no tratamento da Covid-19; estudo não foi divulgado, 2020. Available: https://g1.globo.com/bemestar/coronavirus/noticia/2020/10/19/ministerio-da-ciencia-e-tecnologia-afirma-que-vermifugo-reduz-carga-viral-no-tratamento-precoce-da-covid-19-estudo-nao-foi-revisado-pelos-pares.ghtml [Accessed 25 Feb 2021].

[40] Rocco PRM, Silva PL, Cruz FF, et al. Early use of nitazoxanide in mild COVID-19 disease: randomised, placebo-controlled trial. Eur Respir J 2021;58. 10.1183/13993003.03725-2020. [Epub ahead of print: 08 07 2021]. PMC7758778 33361100

[41] Haneef R, Lazarus C, Ravaud P, et al. Interpretation of results of studies evaluating an intervention highlighted in Google Health News: a cross-sectional study of news. PLoS One 2015;10:e0140889. 10.1371/journal.pone.0140889 26473725 PMC4608738

[42] Fletcher RH, Black B. "Spin" in scientific writing: scientific mischief and legal jeopardy. Med Law 2007;26:511–25. 17970249

[43] John MZ. Reliable knowledge: an exploration of the grounds for belief in science. Cambridge, MA: Cambridge University Press, 1979.

[44] Ziman JM. Public knowledge: an essay concerning the social dimension of science. Cambridge, MA: Cambridge University Press, 1968.

[45] Bavel JJV, Baicker K, Boggio PS, et al. Using social and behavioural science to support COVID-19 pandemic response. Nat Hum Behav 2020;4:460–71. 10.1038/s41562-020-0884-z 32355299

[46] Hiebert RE. Courtier to the crowd: the story of ivy Lee and the development of public relations. Ames, IA: Iowa State University Press, 1966.

[47] Autzen C. Press releases — the new trend in science communication. J Sci Commun 2014;13:C02. 10.22323/2.13030302

[48] Ginger P, Catherine O. EurekAlert! survey confirms challenges for science communicators in the post-print era. Journal of Science Communication 2006;5:1–12.

[49] Weisberg DS, Landrum AR, Hamilton J, et al. Knowledge about the nature of science increases public acceptance of science regardless of identity factors. Public Underst Sci 2021;30:120–38. 10.1177/0963662520977700 33336623

[50] Bantimaroudis P, Kampanellou E. The cultural framing hypothesis: attributes of cultural alliances and conflicts. The International Journal of Press/Politics 2007;12:80–90. 10.1177/1081180X07299793

[51] Haider-Markel DP, Joslyn MR. Gun policy, opinion, tragedy, and blame Attribution: the conditional influence of issue frames. J Polit 2001;63:520–43. 10.1111/0022-3816.00077

[52] Shah DV, Kwak N, Schmierbach M, et al. The interplay of news frames on cognitive complexity. Hum Commun Res 2004;30:102–20. 10.1111/j.1468-2958.2004.tb00726.x

[53] Gorp B. Where is the frame?: Victims and intruders in the Belgian press coverage of the asylum issue. Eur J Commun 2005;20:484–507. 10.1177/0267323105058253

[54] Brosius H-B, Engel D. The causes of third-person effects: unrealistic optimism, impersonal impact, or generalized negative attitudes towards media influence? Int J Public Opin Res 1996;8:142–62. 10.1093/ijpor/8.2.142

[55] Chapin JR. Third-person perception and optimistic bias among urban minority at-risk youth. Communic Res 2000;27:51–81. 10.1177/009365000027001003

[56] Duck JM, Hogg MA, Terry DJ. Social identity and perceptions of media persuasion: are we always less influenced than others?. J Appl Soc Psychol 1999;29:1879–99. 10.1111/j.1559-1816.1999.tb00156.x

[57] Eveland W, Nathanson A, Detenber B. Rethinking the social distance corollary: perceived likelihood of exposure and the third-person perception. Communication Research 1999;26:275–302.

[58] Gunther AC, Chia SC-Y. Predicting pluralistic ignorance: the Hostile media perception and its consequences. Journal Mass Commun Q 2001;78:688–701. 10.1177/107769900107800405

[59] Peiser W, Peter J. Third-Person perception of Television-Viewing behavior. J Commun 2000;50:25–45. 10.1111/j.1460-2466.2000.tb02832.x

[60] Perloff RM. Ego-involvement and the third person effect of televised news coverage. Communic Res 1989;16:236–62. 10.1177/009365089016002004

[61] Perloff RM. Third-person effect research 1983–1992: a review and synthesis. Int J Public Opin Res 1993;5:167–84. 10.1093/ijpor/5.2.167

[62] Rucinski D, Salmon CT. The ‘Other’ as the vulnerable voter: a study of the third-person effect in the 1988 U.S. presidential campaign. Int J Public Opin Res 1990;2:345–68. 10.1093/ijpor/2.4.345

[63] Watts MD, Domke D, Shah DV. Elite cues and media bias in presidential campaigns: explaining public perceptions of a liberal press. Commun Res 1999;26:144–75. 10.1177/009365099026002003

[64] Williams D. Motivated ignorance, rationality, and Democratic politics. Synthese 2021;198:7807–27. 10.1007/s11229-020-02549-8

[65] Taylor L. 'We are being ignored': Brazil's researchers blame anti-science government for devastating COVID surge. Nature 2021;593:15–16. 10.1038/d41586-021-01031-w 33907333

[66] Hallal PC. SOS Brazil: science under attack. Lancet 2021;397:373–4. 10.1016/S0140-6736(21)00141-0 PMC7825897 33493436

